# Association between oral health status and frailty in older adults: a systematic review and meta-analysis

**DOI:** 10.3389/fpubh.2025.1514623

**Published:** 2025-03-31

**Authors:** Jingwen Huang, Yin Zhang, Min Xv, Likai Sun, Mei Wang

**Affiliations:** ^1^Department of Nursing, Tongji Hospital, Tongji Medical College of Huazhong University of Science and Technology, Wuhan, China; ^2^Department of Hospital Infection Control, Tongji Hospital, Tongji Medical College of Huazhong University of Science and Technology, Wuhan, China

**Keywords:** oral health, frailty, older adults, meta-analysis, systematic review

## Abstract

**Aim:**

This study aims to explore the association between oral health status and frailty in older adults using comprehensive and objective oral health measurement indicators.

**Method:**

This study conducted a collection and retrieval of relevant literature in the following English databases: PubMed, Embase, Cochrane Library, and Web of Science (up to July 1, 2024). After screening the literature according to the specified inclusion and exclusion criteria, the quality of the literature was assessed using the Joanna Briggs Institute critical appraisal tool and the Newcastle-Ottawa Scale. The degree of heterogeneity was then represented by the *I*^2^ statistic, and based on this result, a random-effects or fixed-effects model was determined for analysis. Odds ratios (OR)/standardized mean differences (SMD) and 95% confidence intervals were employed to identify the association between various oral health indicators and frailty. Sensitivity analysis was performed for all outcomes.

**Results:**

A total of 28 articles were included. Number of teeth (SMD: −0.591), functional dentition (≥21 teeth) (OR: 0.236), no false teeth (OR: 0.733), ≤20 teeth/with denture (OR: 2.320), tooth brushing daily (OR: 0.562), tongue pressure/decreased tongue pressure (SMD: −0.582/OR: 1.618), occlusal force/occlusal force reduced (SMD: −0.526/OR: 1.846), oral diadochokinesis hypofunction (OR: 1.876), poor mixing ability (OR: 2.303) and oral health assessment tool scores ≥4 (OR: 2.501) were correlated with frailty in older adults.

**Conclusion:**

Various types of oral health indicators are associated with frailty in older adults. Investigating the relationship between oral health and frailty in older adults is of significant importance for preventing frailty, ensuring the quality of life for older adults, and promoting healthy longevity.

**Systematic review registration:**

PROSPERO CRD42024587687.

## Introduction

The world is undergoing a transformation of unprecedented scale in a century, with many countries experiencing a significant acceleration in population aging, marking a rapid global demographic shift (1, 2). The rapid increase in older adults brings forth a suite of health-related issues, among which physical frailty stands as one of the most critical concerns (3, 4). Frailty, once considered a complex clinical entity related to aging, has now transformed into a multifaceted bio-psycho-social syndrome (5, 6). In clinical terms, frailty is perceived as a state of heightened vulnerability, marked by a diminution in the functionality of various bodily systems and a depletion of physiological reserves, thus impacting the capacity to manage everyday and acute stressors effectively (7, 8). In the past decade, as frailty has continued to evolve, it has garnered increasing attention (9). Currently, frailty is recognized as a significant public health issue, imposing a substantial burden of disease on the population, particularly among older adults (10, 11). In a study covering 62 countries worldwide, an estimated prevalence of frailty of up to 12% was reported (11). Furthermore, frailty can lead to a cascade of adverse health outcomes such as cardiovascular disease, falls, hospitalization, disability, and death, causing harm to both mental and physical well-being (12–15). Additionally, Ekram and colleagues explored the association between frailty and all-cause mortality in community older adults (16). In light of the aforementioned research findings, there is an urgent need in the future to prioritize the occurrence of frailty in older adults in order to protect and enhance the quality of life for older adults.

Oral health is a critical determinant of overall health and shares a complex, multifaceted relationship with health, especially for older adults, where it is closely linked to physiological health, happiness, life quality, and self-perception (17, 18). However, as age advances, the burden of oral diseases increases, often leading to poor oral health in older adults, which is connected with various conditions encompassing different aspects of frailty (19, 20). For instance, from a physiological perspective, a study conducted by Xu et al. among the older Chinese population suggests that tooth loss might be closely associated with the occurrence of frailty due to its impact on dietary intake (21). Additionally, Hayashi et al. found a positive correlation between having fewer than 20 natural teeth and the incidence of frailty in a Japanese older female population (22). More importantly, on the psychological front, poor oral health may affect older people’s socialization, the incidence of depression as well as, among other things, mental health and lead to physical frailty (14, 23, 24).

Given the above research background, there is a growing interest in the connection between oral health status and frailty among older adults (25). For instance, a study by Dros in the northern Netherlands found a significant correlation between the deterioration of oral health and frailty in older adults over the age of 55 (26). Kang’s research arrived at similar conclusions, indicating that chewing difficulties were significantly associated with frailty in older adults (27). To date, although several meta-analyses on the relationship between oral health status and frailty in older adults have been reported, they all have certain research gaps. For example, Sakai’s study reported limited oral function indicators that included both qualitative and quantitative data and did not focus on a comprehensive analysis of frailty (28). In Kojima’s study, on one hand, only the relationship between chewing function and frailty was analyzed, and on the other hand, it included only self-reported subjective evaluation data (29).

Therefore, the present study aims to encompass a comprehensive set of objective oral health indicators to analyze the association between oral health and frailty in older adults.

## Method

The protocol for literature retrieval in this study was thoroughly crafted based on the guidelines set forth by the Preferred Reporting Items for Systematic Reviews and Meta-Analyses (PRISMA) in the year 2020 (30).

### Search strategy

For this study, a search was conducted in English electronic databases for relevant literature published up to July 1, 2024, focusing primarily on four databases: PubMed, Embase, Cochrane Library, and Web of Science. The main English search terms utilized in this study were as follows: dental, oral, tooth, teeth, mouth, Carious Lesions, Carious Lesion, Lesion, Carious, Lesions, Carious, Carious Dentin, Carious Dentins, Dentin, Carious, Dentins, Carious, Root Caries, Caries, Root, Caries, Cervical, Cary, Cervical, Cervical Cary, Cervical Caries, Periodontal Diseases, Disease, Periodontal, Diseases, Periodontal, Periodontal Disease, Parodontosis, Parodontoses, Pyorrhea Alveolaris, Periodontitis, Periodontitides, Pericementitis, Pericementitides, Gingivitis, Gingivitides, Xerostomia, Xerostomias, Asialia, Asialias, Hyposalivation, Hyposalivations, Dentures, Denture, Toothbrushing, Toothbrushings, Dentifrices, Dentifrice, Mouthwashes AND Frailty, Frailties, Frailness, Frailty Syndrome, Frailty, Debilities. In addition, in the supporting materials, this study also provides a specific search formula for PubMed (Supplementary Table S1).

### Inclusion and exclusion criteria

The literature screening component of this paper was conducted independently by the authors based on the following inclusion and exclusion criteria. The inclusion criteria established for this study were founded on the Population, Intervention, Comparison, Outcome (PICO) principle, to more accurately identify articles pertinent to this research. Inclusion criteria: (1) Study population: Older adults as defined by the original studies; (2) Four types of oral health indicators: I: Dental-related; II: Oral hygiene care; III: Oral function; IV: Comprehensive oral health score. Specific oral health status indicators and their definitions can be found in Supplementary Table S2. (3) Criteria for the assessment of frailty: I: Cardiovascular Health Study (CHS); II: Japanese version of the CHS (J-CHS); III: Fatigue, Resistance, Ambulation, Illnesses, and Loss of weight (FRAIL) Scale; IV: Frailty Index (FI); V: Kihon Checklist (KCL); VI: Groningen Frailty Indicator (GFI). (4) Type of study: Cross-sectional study, and prospective cohort study. Detailed scoring or definitions of the above six methods of evaluating debilitation are shown in Supplementary Table S3. Exclusion criteria: (1) Experimental animal studies; (2) Reviews, meta-analyses, case reports, conference abstracts, editorial materials, trial registry records, and guidelines; (3) Non-English language literature; (4) Studies with incompatible themes.

### Data extraction

Regarding the data collection process, after sifting through and selecting all the eligible papers, researchers meticulously reviewed the full text of each publication and independently and diligently gathered various data indicators. Below was the data collected from each literature source: Publication year, author, study type, sample size, gender ratio, average age of the study population, methods of frailty assessment, prevalence of frailty, and oral health-related indicators.

### Approaches of literature quality assessment

In the present investigation, different scales were employed to assess the quality of literature for different study types. For cross-sectional studies, the assessment was conducted using the Joanna Briggs Institute (JBI) critical appraisal tool, which encompasses a spectrum of 8 criteria, each offering a binary “Affirmative,” “Negative,” or “Ambiguous” response (31). In the instance of cohort studies, the Newcastle-Ottawa Scale (NOS) was applied, which assigns a cumulative score out of 9, delineating study quality into low (0–3 points), fair (4–6 points), and high (7–9 points) tiers (32). The NOS scale consists of three main entries: “Choice,” “Comparability,” and “Outcome,” and each study can have up to one “*” for each of the entries for “Choice” and “Outcome,” and up to two entries for “Comparability.” Each study can have up to one “*” per entry for “choice” and “outcome” and up to two “*” for “comparability.” “*” for “Comparability” and up to two “*” for “Comparability.” Specific entries for both scales are provided in Supplementary Tables S4, S5.

### Statistical analysis

Continuous data were analyzed employing the standardized mean difference (SMD) as the index of statistical measure, and categorical data were subjected to the odds ratio (OR) as the measure of association. Effect sizes were presented with 95% confidence intervals (CIs). Assessment for heterogeneity was conducted for every outcome metric, utilizing the *I*^2^ statistic; where the *I*^2^ value exceeded the threshold of 50%, analyses were performed using a random-effects model, and conversely, a fixed-effects model was engaged. Furthermore, stratified analyses were executed according to the methodologies of frailty assessment and the regions from which the studies were derived. Sensitivity analyses were applied across all endpoints, and in instances where the corpus of included literature surpassed a count of 10 publications, an examination for publication bias was instituted. The suite of analytical processes was performed utilizing Stata 15.1 software, with statistical significance ascertained at the *p* < 0.05.

## Results

### Results of literature screening

**Figure 1:**
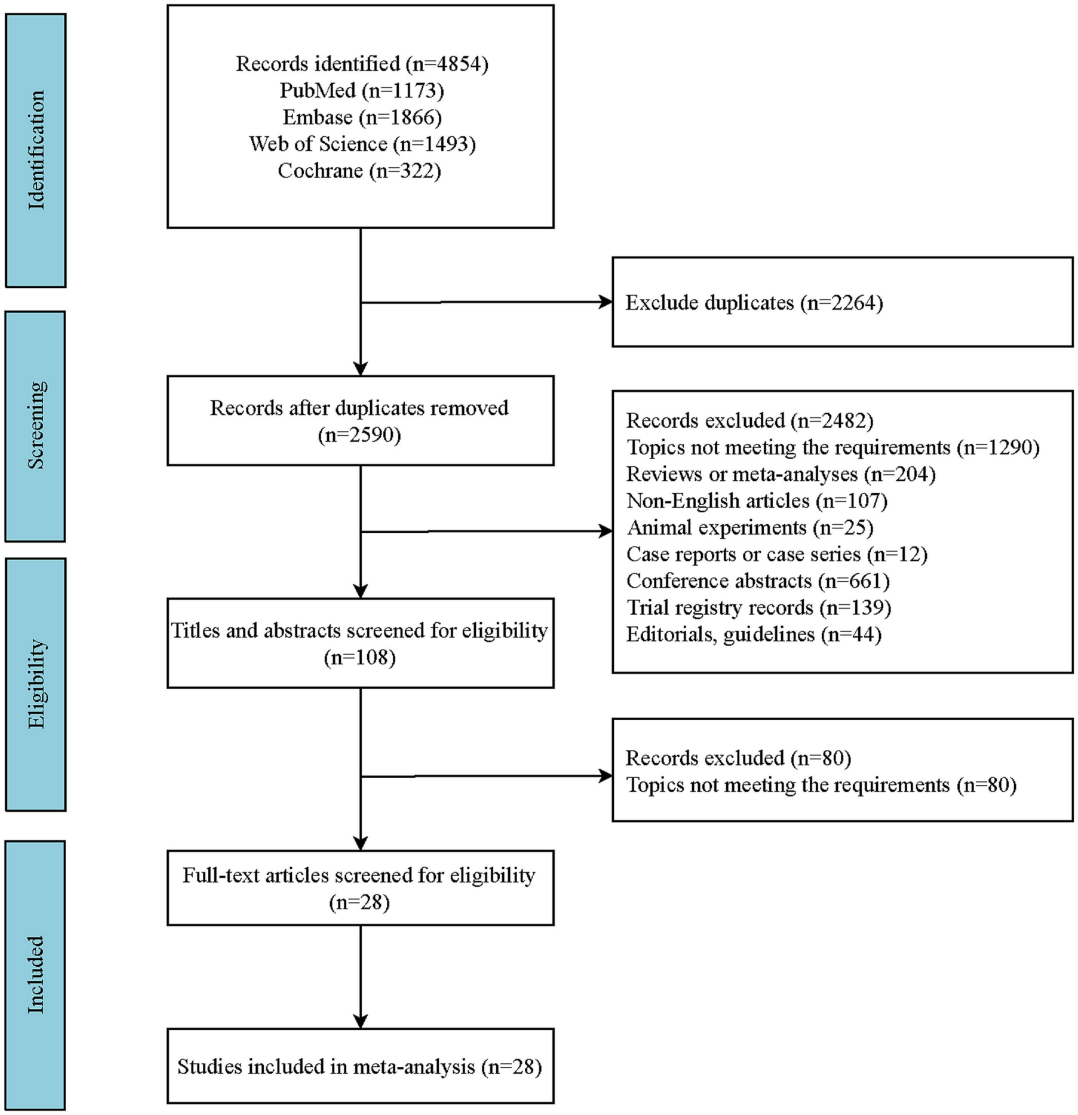
The search flow diagram.

Compliant with the predefined search protocol, a comprehensive search within English-language databases yielded a cumulative total of 4,854 articles. Employing EndNote X20 software for de-duplication, we eliminated 2,264 redundant entries, culminating in a refined pool of 2,590 articles. Subsequently, applying the established criteria for inclusion and exclusion, the final selection encompassed 28 articles (33–60). The specific search process is shown in Figure 1.

### Study characteristics

This investigation ultimately incorporated a curated selection of 28 scholarly works, involving a cumulative total of 36,634 older patients. As depicted in Table 1, the research spanned 11 countries, with the majority (25 studies) being cross-sectional studies, and the remaining 3 were cohort studies. All studies provided pertinent information such as sample size, gender ratio, and average age. Specific baseline data are presented as delineated in Table 1. Besides, Supplementary Table S6 also presents the characteristics of the included primary studies and their respective findings.

**Table 1:** Information on baseline characteristics of included studies.

Author	Year	Country	Study design	Sample size	Male/female	Age (years)	Frailty assessment	Prevalence of frailty, *n* (%)	Oral health characteristics
Xia (56)	2024	China	Cross-sectional multicenter Study	6,664	2,490/4,174	62.23 ± 8.37	J-CHS	413 (6.2)	No false teeth
Wibianty (55)	2024	Indonesia	Cross-sectional study	60	21/39	71.17 ± 9.08	FRAIL scale	28 (46.6)	Functional dentition
Hong (41)	2024	Korea	Cross-sectional study	88	9/79	71.9 ± 5.8	CHS	51 (57.95)	OHAT score
Zhang (59)	2023	China	Cohort study	1,155	572/583	74.93 ± 10.37	FI	265 (22.94)	Functional dentition, brushing frequency
Liu (45)	2023	China	Cross-sectional study	1,280	601/679	77.64 ± 9.87	FRAIL scale	686 (53.6)	OHAT score, brushing frequency
Cruz-Moreira (35)	2023	Ecuador	Cross-sectional study	589	206/383	72 (66, 82)	CHS	170 (28.9)	Occlusal force
Tan (51)	2022	Singapore	Cross-sectional study	1,047	448/599	71.2 ± 5.5	FRAIL scale	65 (6.2)	No false teeth
Ohara (47)	2022	Japan	Cross-sectional study	643	242/401	73.9 ± 6.7	J-CHS	29 (4.5)	ODK hypofunction
Hakeem (24)	2021	United Kingdom	National cross-sectional study	2,368	1,132/1,236	69.1 (69.6, 69.5)	FI	916 (38.7)	Number of teeth
Everaars (37)	2021	The Netherlands	Cross-sectional study	1,202	545/657	72.79 ± 8.07	GFI	222 (18)	No false teeth
Zhang (60)	2020	China	Cross-sectional study	4,037	1,687/2,350	67.8 ± 5.9	J-CHS	270 (6.7)	Functional dentition
Valdez (52)	2020	Australia	Cross-sectional study	601	601/0	≥70	CHS	116 (19.3)	Periodontal disease
Hakeem (38)	2020	United Kingdom	Cross-sectional study	356	257/99	67.13 ± 6.5	FI	155 (43.5)	Number of teeth, functional dentition
Satake (48)	2019	Japan	Cross-sectional study	467	294/173	69.36 ± 6.84	FRAIL scale	47 (10.1)	Number of teeth, periodontal disease, tongue pressure, ODK hypofunction
Hasegawa (40)	2019	Japan	Cross-sectional study	308	107/201	72.7 ± 7.1	KCL	20 (6.5)	Oral moisture
Iwasaki (43)	2018	Japan	Cross-sectional study	141	40/101	72 (66, 78)	CHS	32 (22.7)	Number of teeth, no false teeth
Watanabe (54)	2017	Japan	Cross-sectional study	4,720	2,274/2,446	72.1 ± 5.6	CHS	535 (11.3)	Number of teeth, occlusal force
Andrade (36)	2013	Brazil	Cross-sectional study	1,374	554/820	≥60	CHS	117 (8.5)	Functional dentition, no false teeth
Castrejón-Pérez (33)	2012	México	Cross-sectional study	699	327/372	77.9 ± 6.3	CHS	105 (15)	Number of teeth
Yoshida (58)	2022	Japan	Cross-sectional study	340	69/271	78.4 ± 5.2	CHS	34 (10.3)	Oral moisture, occlusal force, tongue pressure, masticatory function
Nakamura (46)	2021	Japan	Cross-sectional study	832	303/529	74.9 ± 6.29	CHS	44 (5.5)	Oral dryness, ODK hypofunction, tongue pressure
Watanabe (53)	2020	Japan	Cross-sectional study	1,106	548/558	73.38 ± 5.38	J-CHS	130 (11.75)	Poor mixing ability
Shimazaki (49)	2020	Japan	Cross-sectional study	973	463/510	Male, 73.2 (69.0, 77.0); Female, 73.2 (69.0, 77.0)	KCL	88 (9.04)	Oral dryness, ODK hypofunction, tongue pressure
Horibe (42)	2018	Japan	Cross-sectional study	659	264/395	73.16 ± 5.59	FRAIL scale	92 (14.0)	Poor mixing ability
Yamanashi (57)	2018	Japan	Cross-sectional study	1,603	650/953	72.8 ± 7.4*	CHS	30 (1.9)	Tongue pressure
Kimble (44)	2023	United Kingdom	Cross-sectional study	BRHS, 1013; HABC, 1975	BRHS, 1013/0; HABC, NR	BRHS, 85 ± 4; HABC, 75 ± 3	CHS	BRHS, 182 (17.97); HABC, 97 (4.9)	Functional dentition, no false teeth
Castrejón-Pérez (34)	2017	México	Cohort study	237	115/122	76.4 ± 5.2	CHS	35 (14.8)	Number of teeth
Takeuchi (50)	2022	Japan	Cohort study	97	34/63	71.9 ± 5.4	J-CHS	34 (35.05)	Oral moisture, tongue pressure, occlusal force, masticatory function

FI, Frailty index; FRAIL, Fatigue, resistance, ambulation, illnesses, and loss of weight; GFI, Groningen Frailty Indicator; CHS, Cardiovascular Health Study; J-CHS, Japanese version of the CHS; KCL, Kihon Checklist; OHAT, Oral Health Assessment Tool; ODK, Oral diadochokinesis; BRHS, British Regional Heart Study; HABC, Health, Aging, and Body Composition.

### Literature quality assessment

In Supplementary Table S4, the number of entries from cross-sectional studies that met the JBI checklist criteria was displayed. This study found that all included literature met four or more criteria, demonstrating adequate methodological quality. As shown in Supplementary Table S5, among the three cohort studies, two were rated as moderate quality (scoring 6 points each), and one was rated as high quality (scoring 7 points).

### Results of the analysis of the relationship between indicators related to oral teeth and frailty

#### Number of teeth

**Figure 2:**
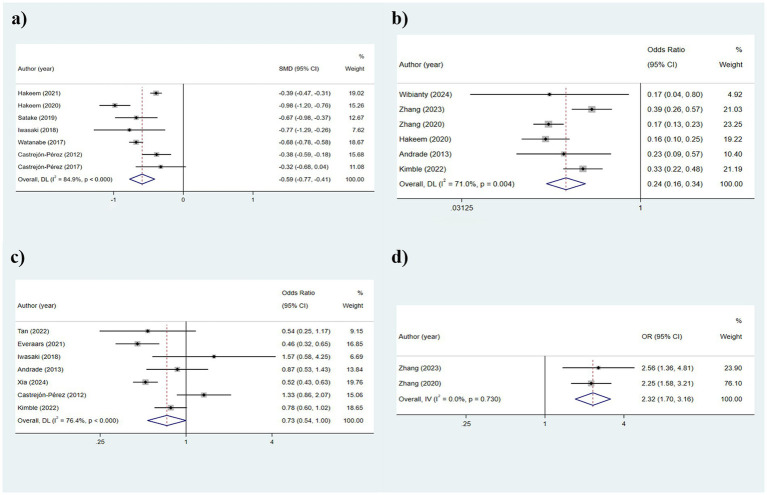
Results of the analysis of the relationship between indicators related to oral teeth and frailty: **(a)** Number of teeth; **(b)** Functional dentition (≥21 teeth); **(c)** No false teeth; **(d)** ≤20 teeth/with denture.

**Figure 3:**
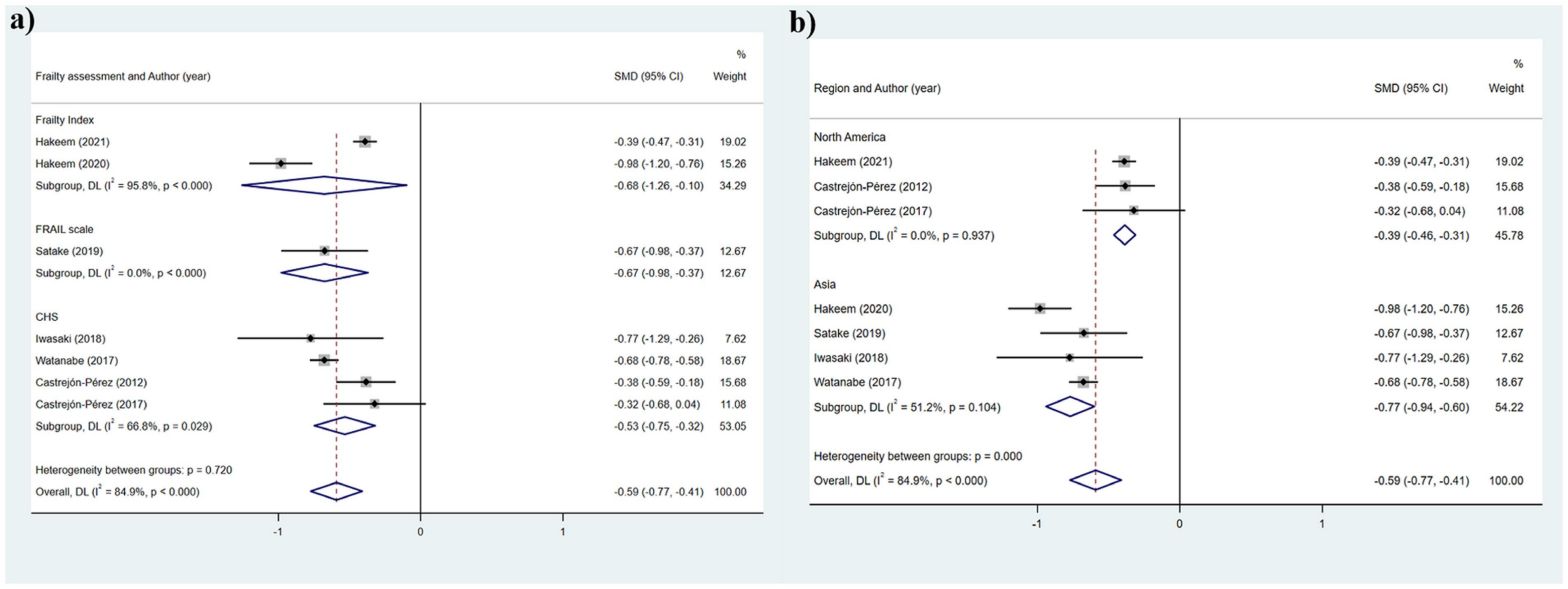
Subgroup analysis of the relationship between number of teeth and frailty: **(a)** Frailty assessment criteria; **(b)** Geographical regions.

Table 2 illustrates that seven studies were incorporated into this analysis. The assessment of heterogeneity determined *I*^2^ = 84.9%, which justified the application of a random-effects model. The findings indicated that there was an association between dental count and the state of frailty (SMD: −0.591; 95%CI: −0.772, −0.411; *p* < 0.001) (Figure 2a). Additionally, subgroup analyses were conducted to explore the sources of heterogeneity. Initially, subgroup analyses were performed based on three frailty assessment criteria: the FRAIL scale, CHS, and FI. The results were found to be consistent with the aforementioned findings [FRAIL scale: SMD: −0.675, 95%CI: −0.980, −0.370; CHS: SMD: −0.533, 95%CI: −0.747, −0.319; FI: SMD: −0.677, 95%CI: −1.256, −0.099; all *p* < 0.001] (Figure 3a). Subsequently, stratified analyses were conducted according to two geographical regions, Asia and North America, and the results remained consistent [Asia: SMD: −0.770, 95%CI: −0.940, −0.600; North America: SMD: −0.387, 95%CI: −0.463, −0.311; all *p* < 0.001] (Figure 3b).

**Table 2:** Results of the association analysis between four types of oral health status indicators and frailty.

Indicators for classification of outcomes	Analysis indexes		Number of studies	SMD/OR (95%CI)	*p*	*I*^2^ (%)
**Dental-related indicators**						
Number of teeth	Overall		7	−0.591 (−0.772, −0.411)	<0.001	84.9
	Subgroup analysis					
	Frailty assessment	FRAIL scale	1	−0.675 (−0.980, −0.370)	<0.001	0.0
		FI	2	−0.677 (−1.256, −0.099)	0.022	95.8
		CHS	4	−0.533 (−0.747, −0.319)	<0.001	66.8
	Region	Asia	4	−0.770 (−0.940, −0.600)	<0.001	51.2
		North America	3	−0.387 (−0.463, −0.311)	<0.001	0.0
Functional dentition (≥21 teeth)	Overall		6	0.236 (0.162, 0.344)	<0.001	71.0
	Subgroup analysis						Frailty assessment	FRAIL scale	1	0.171 (0.037, 0.798)	0.025	0.0		FI	2	0.251 (0.104, 0.605)	0.002	88.1		CHS	2	0.310 (0.218, 0.442)	<0.001	0.0		J-CHS	1	0.168 (0.125, 0.226)	<0.001	0.0	Region	Asia	5	0.216 (0.139, 0.335)	<0.001	70.0		Europe	1	0.328 (0.224, 0.481)	<0.001	0.0
	No false teeth	Overall		7	0.733 (0.538, 1.000)	0.050	76.4
		Subgroup analysis						Frailty assessment	FRAIL scale	1	0.539 (0.248, 1.173)	0.119	0.0		GFI	1	0.457 (0.321, 0.649)	<0.001	0.0		CHS	4	0.981 (0.719, 1.339)	0.904	44.5		J-CHS	1	0.519 (0.425, 0.634)	<0.001	0.0	Region	Asia	4	0.697 (0.455, 1.067)	0.096	60.3		Europe	2	0.605 (0.358, 1.024)	0.061	82.6		North America	1	1.334 (0.861, 2.066)	0.198	0.0
		≤20 teeth, with denture	Overall		2	2.320 (1.703, 3.160)	<0.001	0.0
		**Oral hygiene care related**						
		Tooth brushing daily	Overall		2	0.562 (0.396, 0.797)	0.001	38.4
		**Indicators related to oral function**						
Oral moisture		Overall		3	0.214 (−0.067, 0.495)	0.136	0.0
Oral dryness (<27)		Overall		2	1.141 (0.594, 2.192)	0.693	65.3
Tongue pressure (kPa)		Overall		4	−0.582 (−1.023, −0.141)	0.010	78.0	Subgroup analysis						Frailty assessment	FRAIL scale	1	−0.649 (−0.953, −0.344)	<0.001	0.0		CHS	2	−0.922 (−1.304, −0.540)	<0.001	18.8		J-CHS	1	0.000 (−0.417, 0.417)	1.000	0.0
	Decreased tongue pressure (<30 kPa)	Overall		2	1.618 (1.116, 2.346)	0.011	0.0
	Occlusal force (N)	Overall		3	−0.526 (−0.808, −0.245)	<0.001	62.4	Subgroup analysis						Frailty assessment	J-CHS	1	−0.312 (−0.614, −0.010)	0.043	0.0		CHS	2	−0.674 (−0.774, −0.574)	<0.001	0.0
		Occlusal force reduced	Overall		2	1.846 (1.208, 2.820)	0.005	0.0
		Masticatory function (mg/dl)	Overall		2	−0.442 (−1.026, 0.142)	0.138	55.5
ODK hypofunction		Overall		4	1.876 (1.334, 2.639)	<0.001	0.0
Poor mixing ability	Overall		2	2.303 (1.692, 3.134)	<0.001	25.5
**Comprehensive oral health score**						
OHAT scores ≥ 4	Overall		2	2.501 (1.752, 3.570)	<0.001	0.0

CHS, Cardiovascular health study; CI, Confidence interval; FI, Frailty index; GFI, Groningen frailty indicator; FRAIL, Fatigue, resistance, ambulation, illnesses, and loss of weight; mg/dl, Milligrams per deciliter; J-CHS, Japanese version of the CHS; kPa, Kilopascal; ODK, Oral diadochokinesis; OHAT, Oral health assessment tool; OR, Odds ratio; SMD, Standardized mean difference.

#### Functional dentition (≥21 teeth)

**Figure 4:**
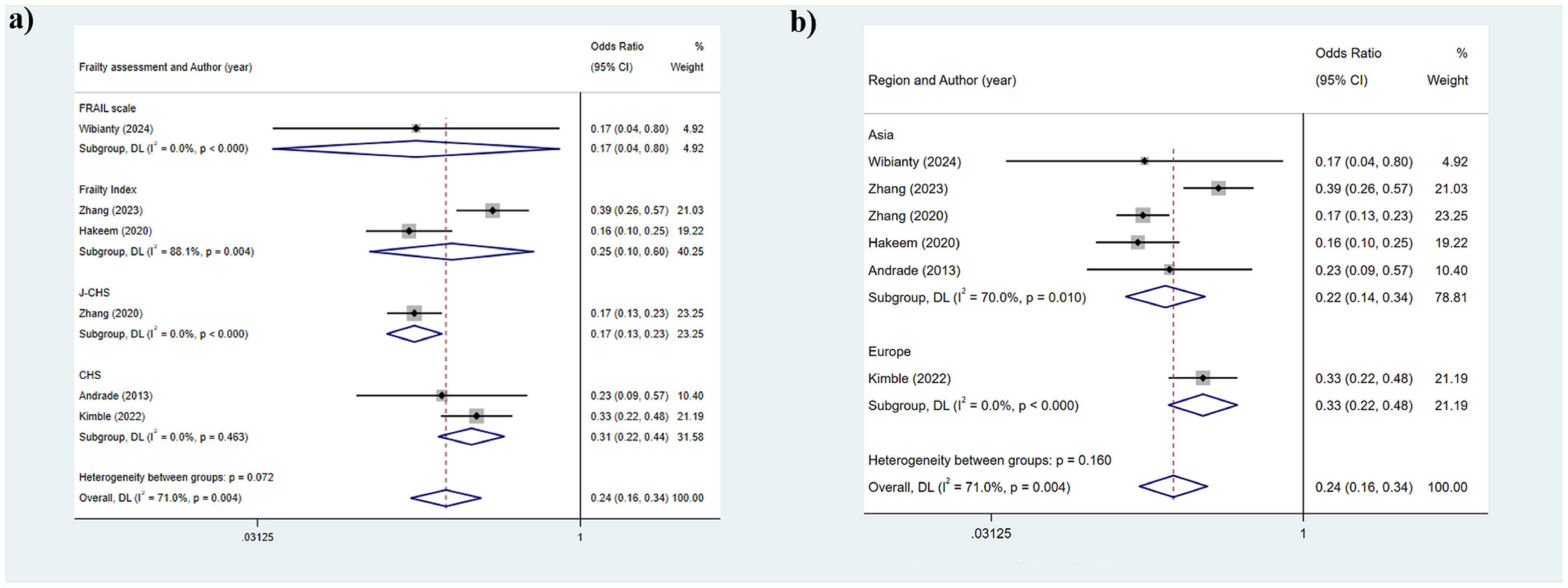
Subgroup analysis of the relationship between functional dentition (≥21 teeth) and frailty: **(a)** Frailty assessment criteria; **(b)** Geographical regions.

The heterogeneity results were analyzed using a random-effects model (*I*^2^ = 71.0%). The findings suggested that functional tooth count was correlated with frailty. Older adults with a functional tooth count <21 had a risk of frailty that was 0.236 times that of those with a functional tooth count ≥21 (95%CI: 0.162, 0.344) (Figure 2b). The results of the subgroup analysis based on the assessment method of frailty were depicted in Figure 4a [FRAIL scale: OR: 0.1721, 95%CI: (0.218, 0.442); CHS: OR: 0.310, 95%CI: (0.218, 0.442); FI: OR: 0.251, 95%CI: (0.104, 0.605); J-CHS: OR: 0.168, 95%CI: (0.125, 0.226)]. The subgroup analysis results based on the study area are depicted in Figure 4b [Asia: OR: 0.328, 95%CI: (0.224, 0.481); Europe: OR: 0.328, 95%CI: (0.224, 0.481)]. The results of the two aforementioned group analyses both exhibited statistical significance, indicating that functional tooth count was related to frailty (all *p* < 0.05).

#### No false teeth

**Figure 5:**
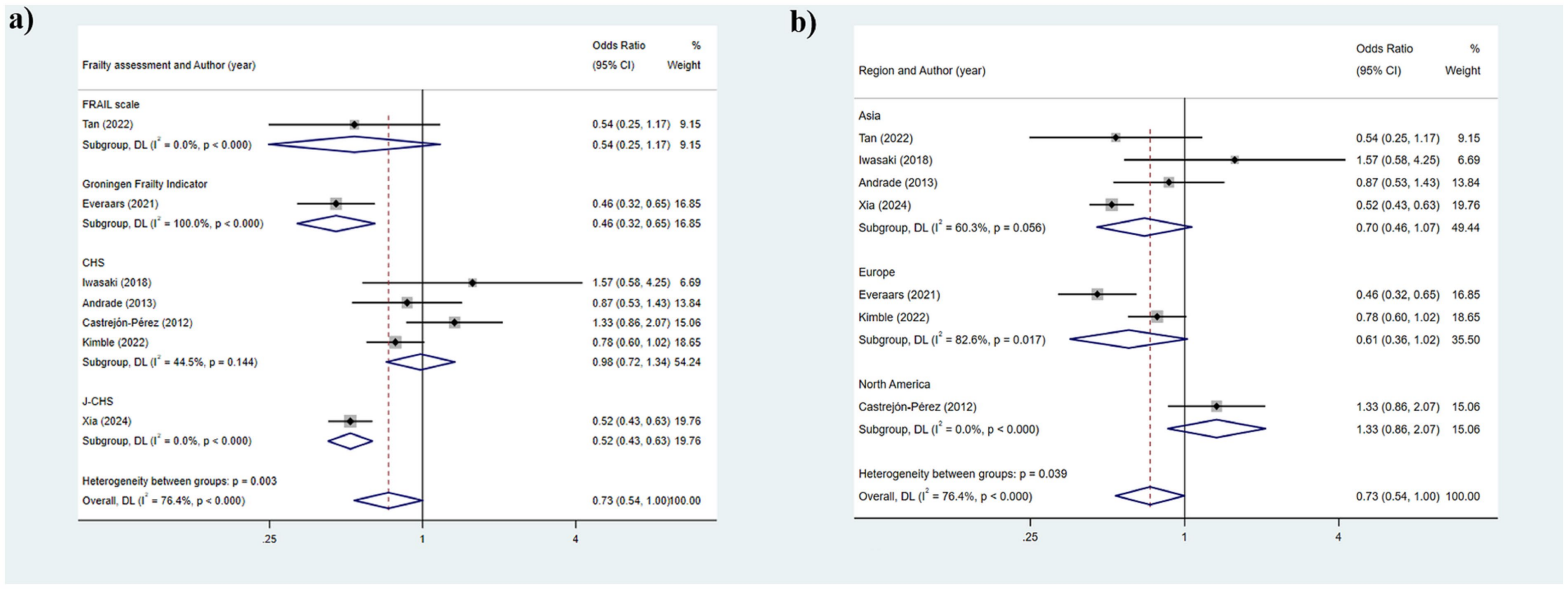
Subgroup analysis of the relationship between no false teeth and frailty: **(a)** Frailty assessment criteria; **(b)** Geographical regions.

The heterogeneity remained relatively high, leading to the adoption of a random-effects model (*I*^2^ = 76.4%). The results suggested that the use of dentures was correlated with frailty (*p* = 0.050) (Figure 2c). Due to the high heterogeneity, subgroup analyses were conducted. The stratified analysis based on frailty assessment methods revealed that the results from the GFI and J-CHS groups were consistent with the overall findings [GFI: OR: 0.457, 95%CI: (0.321, 0.649); J-CHS: OR: 0.519, 95%CI: (0.425, 0.634); all *p* < 0.001], whereas the CHS and FRAIL scale groups showed inconsistencies with the general results [CHS: OR: 0.981, 95%CI: (0.719, 1.339); FRAIL scale: OR: 0.539, 95%CI: (0.248, 1.173); all *p* > 0.05] (Figure 5a). Additionally, subgroup analyses in Asia (OR: 0.698, 95%CI: 0.455, 1.067, *p* = 0.096), North America (OR: 1.334, 95%CI: 0.861, 2.066, *p* = 0.198), and Europe (OR: 0.605, 95%CI: 0.358, 1.024, *p* = 0.198) did not yield consistent outcomes (Figure 5b). This indicated that different frailty assessment methods and study regions were sources of heterogeneity.

#### ≤20 teeth/with denture

This section includes two studies that utilized a fixed-effects model for analysis (*I*^2^ = 0.0%). As illustrated in Figure 2d, the results demonstrated that the risk of frailty in older adults with ≤20 teeth or using dentures was 2.320 times (95%CI: 1.703, 3.160) that of older adults without such conditions (*p* < 0.001).

### Results for analysis of the association between oral hygiene care-related indicators and frailty

#### Tooth brushing daily

**Figure 6:**
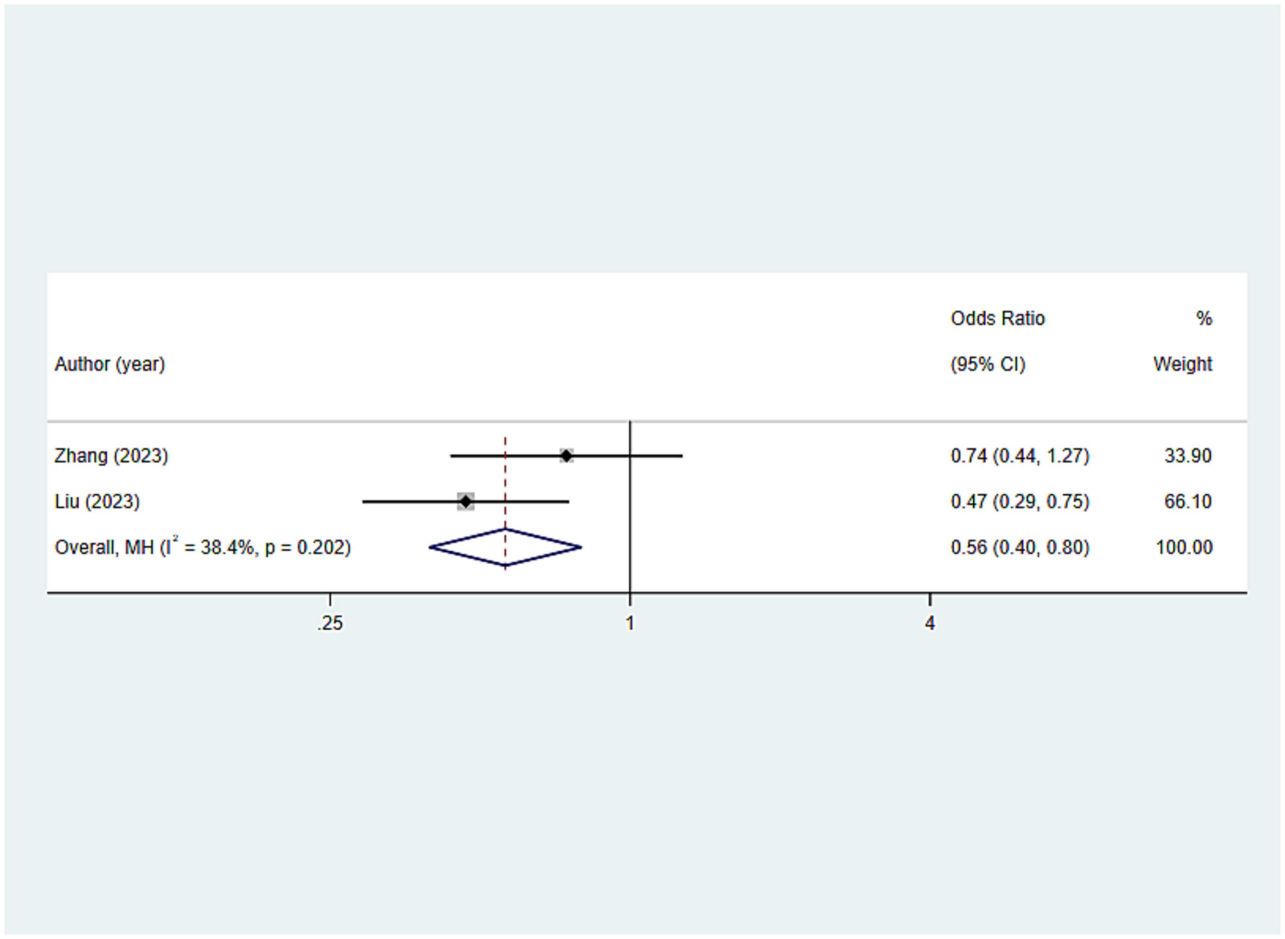
Results for analysis of the association between oral hygiene care-related indicators (tooth brushing daily) and frailty.

This study employed the frequency of daily tooth brushing as an indicator of oral hygiene care. As shown in Figure 6, older adults with a lower frequency of daily tooth brushing were more susceptible to frailty seemingly (OR: 0.562; 95%CI: 0.396, 0.797; *p* = 0.001).

### Outcomes of the relationship between oral function indicators and frailty

#### Oral moisture

**Figure 7:**
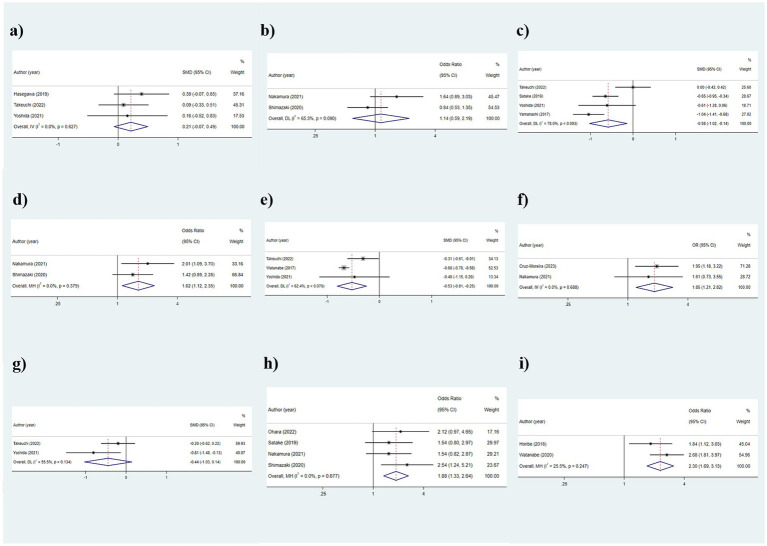
Outcomes of the relationship between oral function indicators and frailty: **(a)** Oral moisture; **(b)** Oral dryness (< 27); **(c)** Tongue pressure; **(d)** Decreased tongue pressure (< 30 kPa); **(e)** Occlusal force (N); **(f)** Occlusal force reduced; **(g)** Masticatory function; **(h)** ODK hypofunction; **(i)** Poor mixing ability.

The analysis results regarding the association between oral moisture and frailty were as follows: SMD: 0.214, 95%CI: (−0.067, 0.495), *p* = 0.136, suggesting no statistically significant association between oral moisture and frailty (Figure 7a).

#### Oral dryness (<27)

As the results were shown in Table 2 and Figure 7b, there was no statistically significant difference in the oral dryness condition between the frailty and non-frailty groups [OR: 1.141, 95%CI: (0.594, 2.192), *p* = 0.693].

#### Tongue pressure

**Figure 8:**
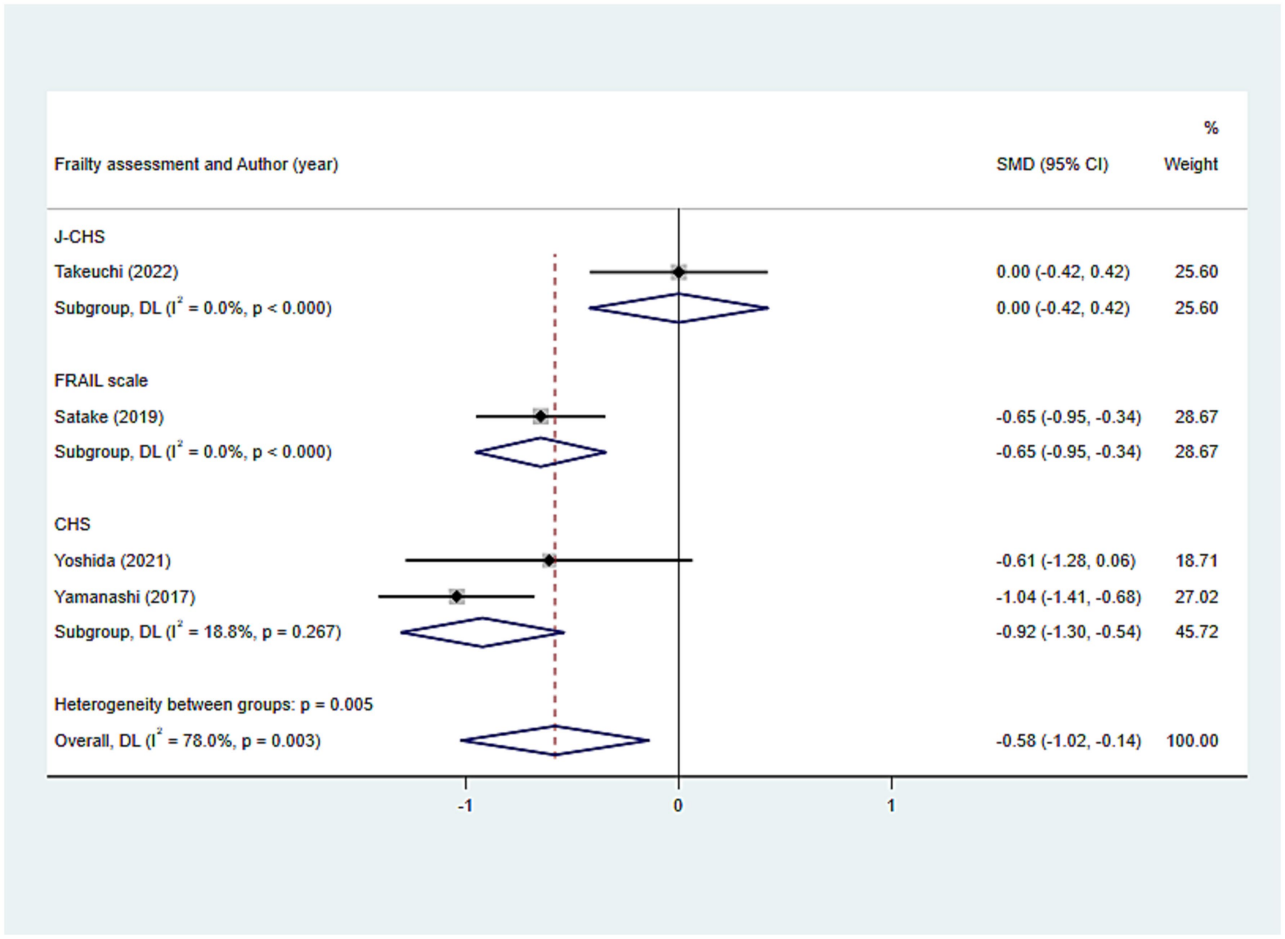
Subgroup analysis of the relationship between tongue pressure and frailty: Frailty assessment criteria.

The outcomes of the random-effects model evaluation were as follows: The value of SMD was −0.582 (95%CI: −1.023, −0.141, *p* = 0.010), indicating that the level of tongue pressure in older adults was related to frailty (Figure 7c). Given the significant heterogeneity (*I*^2^ = 78.0%), subgroup analyses were conducted based on different frailty assessment methods, with the FRAIL scale and CHS groups showing consistent outcomes (all *p* < 0.001), whereas the J-CHS group did not (*p* = 1.000), suggesting that the diversity in assessment methods accounted for the heterogeneity (Figure 8).

#### Decreased tongue pressure (<30 kPa)

The results, as depicted in Figure 7d, demonstrated that older adults with decreased tongue pressure (<30 kPa) were at a higher risk of developing frailty [OR: 1.618, 95%CI: (1.116, 2.346), *p* = 0.011].

#### Occlusal force (N)

**Figure 9:**
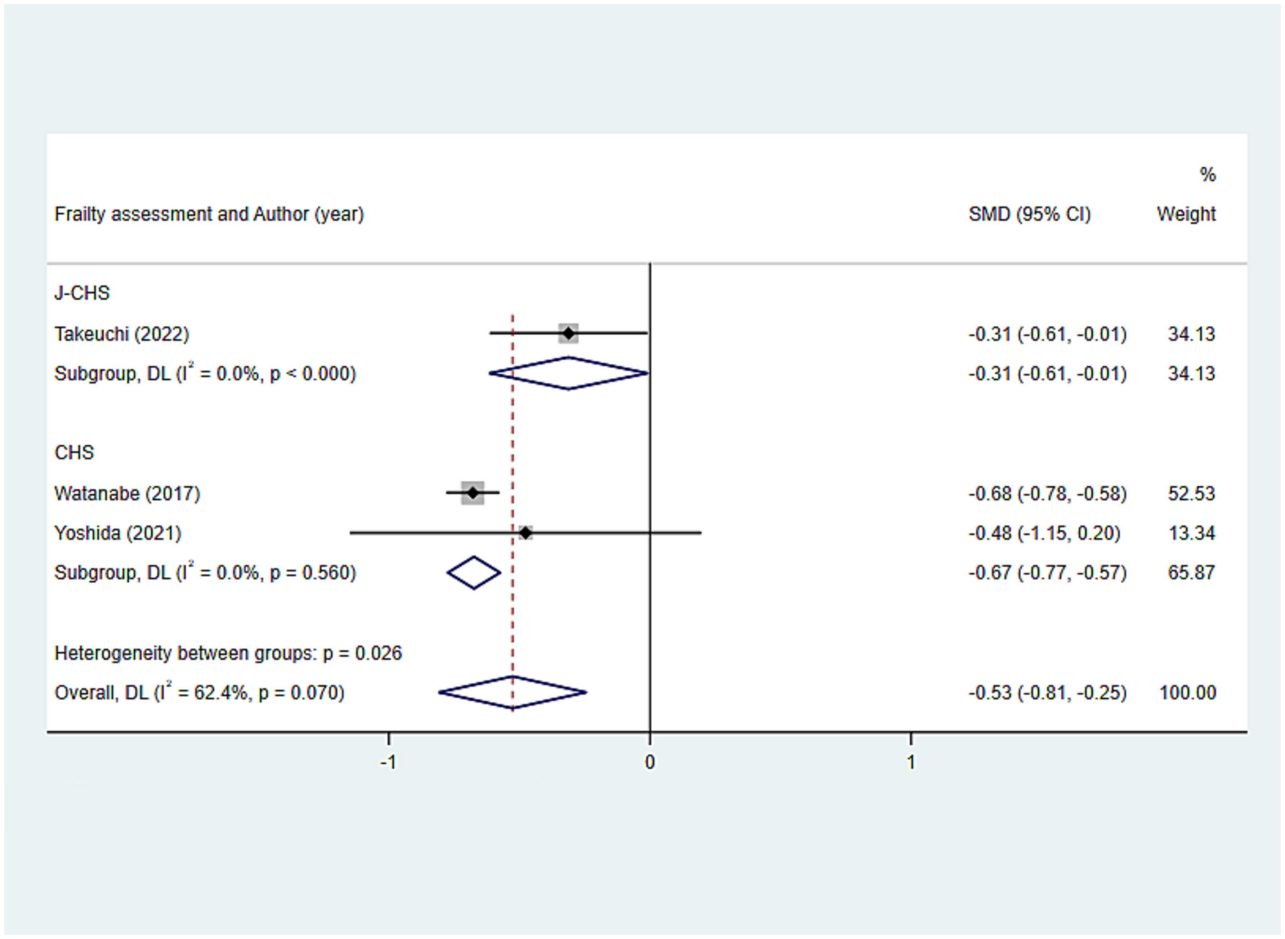
Subgroup analysis of the relationship between occlusal force and frailty: Frailty assessment criteria.

The findings of the relationship between occlusal force and frailty were presented below: SMD: −0.526, 95%CI: (−0.808, −0.245), *p* < 0.001, assuming that the occlusal force in the frail group was lower than that in the non-frail group, and that occlusal force was associated with frailty (Figure 7e). Additionally, subgroup analyses conducted according to the frailty assessment criteria were consistent with the aforementioned results, indicating a relatively stable outcome [CHS: SMD: −0.674, 95%CI: (−0.774, −0.574); J-CHS: SMD: −0.312, 95%CI: (−0.614, −0.010); all *p* < 0.05] (Figure 9).

#### Occlusal force reduced

The examination of the link between occlusal force reduced and frailty revealed that a reduction in bite force could be associated with frailty, making older adults with occlusal force reduced more prone to experiencing frailty [OR: 1.846, 95%CI: (1.208, 2.820), *p* = 0.005] (Figure 7f).

#### Masticatory function

As shown in Table 2 and Figure 7g, the masticatory function of the fateful group and the non-fateful group could not be considered to have significant statistical significance (*p* = 0.138).

#### Oral diadochokinesis hypofunction

A total of 4 papers were included in this section for analysis, and the results were as follows: OR: 1.876, 95%CI: (1.334, 2.639), *p* < 0.001, assuming that the probability of oral diadochokinesis (ODK) hypofunction was higher in the frailty group than in the non- frailty group and that ODK hypofunction was correlated with frailty (Figure 7h).

#### Poor mixing ability

The results, as shown in Figure 7i, indicate that the risk of frailty in older adults with poor oral mixing ability was 2.303 times (95%CI: 1.752, 3.570) higher than in older adults without this condition.

### Relationship between comprehensive oral health score and frailty

#### Oral health assessment tool scores ≥4

**Figure 10:**
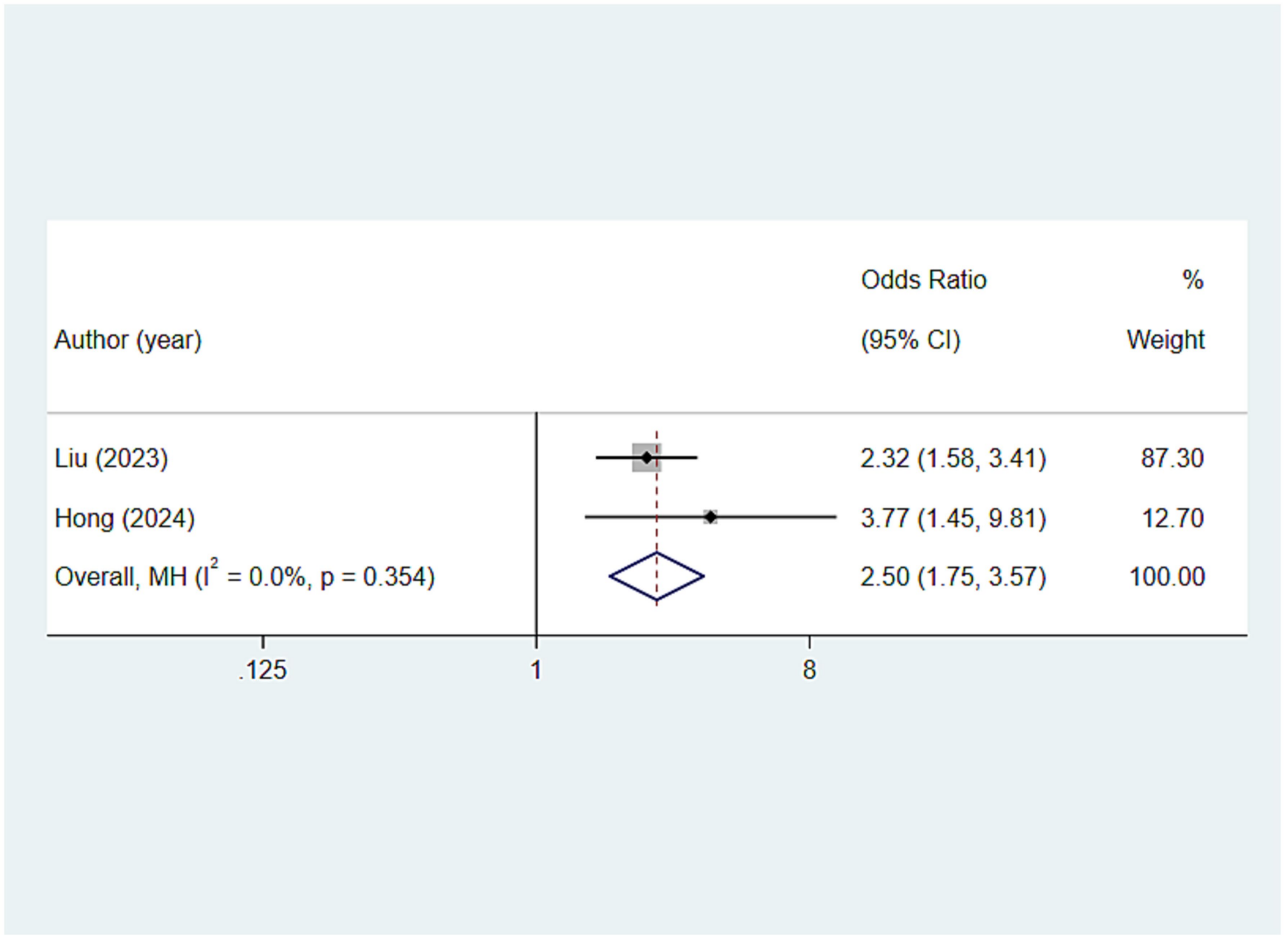
Relationship between comprehensive oral health score (OHAT scores) and frailty.

In the analysis of the relationship between the oral health assessment tool (OHAT) composite score and frailty, this study found that older adults with an OHAT score of ≥4 seem to be at a higher risk of developing frailty [OR: 2.501, 95%CI: (1.752, 3.570), *p* < 0.001] (Figure 10).

### Sensitivity analysis

In the present study, sensitivity analyses were conducted on the associations between oral health status response indicators and frailty under each of the four classifications, as shown in Supplementary Table S7, and the results of the studies were generally similar after excluding any individual study. The above outcomes indicated that the results of the statistical analyses in this study were relatively stable and reliable.

## Discussion

Frailty is highly prevalent among older adults, leading to adverse outcomes, affecting the quality of life for older adults, and posing challenges to the sustainability of the healthcare system (10). Besides, older people often have more oral health issues compared to younger people, which can cause harm to both physical and mental well-being (19, 20). Therefore, based on four types of oral health indicators, this paper comprehensively explores the relationship between oral health and frailty in older adults, with the following key findings: (1) Dental-related indicators: Number of teeth, functional dentition (≥21 teeth), no false teeth and ≤20 teeth/with a denture were associated with frailty; (2) Oral hygiene care: Lower brushing frequency was related to frailty; (3) Oral function: The relationship between tongue pressure/decreased tongue pressure, occlusal force/occlusal force reduced ODK hypofunction, and poor mixing ability were associated with frailty, while no significant statistical association has been found between oral moisture, oral dryness, and masticatory function with frailty; (4) Comprehensive oral health score: OHAT scores ≥4 was correlated with frailty.

Older adults who have experienced tooth loss might frequently be at risk of frailty due to inadequate nutritional intake (61). Additionally, the loss of teeth can lead to social barriers, causing psychological distress that increases the likelihood of developing depression (62, 63). This, in turn, causes older adults to resist social interactions, thereby reducing their level of physical activity, which can ultimately result in frailty (64, 65). The results of the present study on oral dental health indicators support the above conclusions. Consistent with Zhang et al.’s study on the association between the number of teeth and frailty in older adults’ care homes, our research found that older adults in the frail group had fewer teeth than those in the non-frail group (66). Moreover, subgroup analyses conducted based on frailty assessment methods or study regions both suggest that the number of teeth was conceivably associated with frailty. The term “functional dentition” refers to the minimum number of natural teeth required to provide adequate oral function, albeit not under ideal conditions (67). In this investigation, a lower functional dentition in older adults was likely to be associated with an increased risk of frailty, possibly due to the compromised nutritional value of the diet that results from a reduced functional dentition. When exploring the relationship between wearing dentures and frailty, the present investigation, in line with the research by Zhang and colleagues, indicated that older adults without dentures were potentially more susceptible to progressing toward frailty (59). This suggests that properly wearing dentures could potentially help older adults prevent frailty. The above outcomes could be attributed to the fact that the use of dentures in older adults can improve masticatory function, leading to a more varied diet and enhanced nutritional intake, as well as addressing speech difficulties and boosting confidence, which fosters social engagement (68, 69). Finally, this investigation also perceived that ≤20 teeth, with dentures may be associated with the onset of frailty. The aforementioned findings suggested that even with the presence of prosthetic teeth, a lower count of natural teeth can still predispose an individual to frailty, which is consistent with the research outcomes of Kimble et al. (44) and Zhang et al. (60). Therefore, when examining the relationship between dental health and frailty, it is not sufficient to draw conclusions based solely on one or two indicators. Instead, a comprehensive exploration of the association between dental health and frailty, incorporating multiple indicators, is required. The above approach can help establish effective frailty prevention strategies for older adults from the perspective of protecting dental health.

In the realm of oral hygiene care, the research revealed that older adults with a lower frequency of daily tooth brushing were perhaps likely to be at a higher risk of developing frailty, which was in line with the findings of Zhang: Older adults who brushed their teeth regularly were less likely to be frail compared to those who do not brush their teeth, demonstrating that regular tooth brushing may help reduce the risk of frailty (66). However, due to the lack of comprehensive information in this survey, more indicators related to oral hygiene care were not included. Therefore, future research can explore the association between oral health care and frailty further, with the aim of proposing additional preventive measures from a public health perspective to safeguard the physical and mental well-being of older adults.

Almost seven categories of oral function indicators were used to analyze the relationship between oral health and frailty in this investigation. Contrasting with Sakai’s research but aligning with the study by Tanaka et al., our study found that in older adults, reduced tongue pressure/low tongue pressure may be related to frailty (28, 70). This could be due to the fact that decreased tongue pressure is believed to lead to a decline in intake and swallowing functions, resulting in malnutrition and inadequate nutritional intake, thereby affecting the onset of frailty in older adults (48). However, there are evident conflicts in the conclusions of the aforementioned studies, and future research necessitates larger-scale longitudinal studies to substantiate these findings. Next, occlusal force/reduced occlusal force was also revealed to be possibly associated with frailty in this survey, which is consistent with Nakamura’s research (46). Anterior tongue movements reflected in ODK hypofunction are oral functions that play a key role in effective eating and maintaining adequate nutrient intake (71). In our research, ODK hypofunction was shown to possibly correlate with frailty, a finding that was consistent with the research conducted by Tani et al. However, Satake’s research did not corroborate this association (48, 72). Variations in study populations and sample sizes may cause differences. Once more, a relationship between poor mixing ability and frailty was observed, which was consistent with Horibe’s study, revealing that mixing ability was associated with frailty progression (73). Consequently, regarding oral moisture, oral dryness, and masticatory function, a statistical association with frailty was not demonstrated. However, Dibello’s research indicated that these three indicators possessed a certain correlation with frailty (17). We speculate that the reason for the discrepancy might be due to the different frailty assessment methods, different definitions of the study populations in the involved literature, and the lack of stratified analysis. Hence, more extensive and comprehensive research is needed in the future to explore the specific causal relationship between the aforementioned indicators and frailty.

Ultimately, OHAT was employed in the study, a structured and effective method for oral health assessment that has been validated as suitable for non-dental healthcare professionals to evaluate the oral health of older adults (74). The tool was utilized to reflect the overall state of oral health and explore its relationship with frailty in older adults. Rapp’s study has indicated a significant correlation between OHAT scores and frailty, and a similar finding was reported by Kuo et al. (75) and Rapp et al. (76). Their research among older adults in rural China, suggested that OHAT scores can predict frailty. Both of these research conclusions were likewise the findings of the current survey, which also demonstrated that an OHAT score ≥ 4 was possibly associated with frailty. Nonetheless, aside from the OHAT, there are several other oral health evaluation tools capable of providing a comprehensive reflection of the oral health status of older adults, such as the Brief Oral Health Status Examination (BOHSE) and the Oral Assessment Guide (OAG) (77, 78). Different assessment tools perhaps yield varying evaluation outcomes. This study only explored the OHAT, which presents certain limitations, and future researchers might consider focusing on this aspect.

In the preliminary survey, the articles included in this study exhibited substantial variation in terms of geographic region and frailty assessment methods, which may suggest potential confounding factors. To ensure the robustness of the study results, we conducted subgroup analyses for different outcomes based on region and assessment methods. The findings revealed that the results of most subgroups were consistent with the overall results, further demonstrating the stability of the study. Moreover, the factors influencing the occurrence of frailty in older patients are diverse. For instance, age, body weight, and educational level are all associated with frailty in older patients (79). Additionally, the study by Liang et al. indicated that malnutrition was closely related to frailty in patients aged 65 years or older (80). The overall health status or underlying medical conditions of older adults are also potential risk factors for frailty (81, 82). As mentioned above, there are confounding factors that may affect the relationship between oral health and frailty in the older adult. However, due to limitations in the included primary studies, we were unable to extract additional relevant data for meta-analysis. Taken together, these observations highlight the need for future research that can better control for confounding factors, such as prospective cohort studies or randomized controlled trials, to investigate the impact of various factors on frailty.

Within the context of existing research, to gain a more comprehensive and objective understanding of the relationship between oral health and frailty in older adults to a certain extent, this study endeavored to search for and include a greater number of objectively measured oral health indicators to explore their connection with frailty. We hope that this study will contribute to urging older adults to take oral diseases seriously to prevent frailty, encourage clinicians to incorporate more oral health-related indicators in future medical check-ups, and achieve sustainability in the healthcare system. This study has certain strengths: Firstly, it utilizes objective oral health indicators. Secondly, despite the issue of high heterogeneity among studies, subgroup analyses were conducted to trace the sources in all high-heterogeneity analyses. However, like other studies, our analysis also has some limitations. Firstly, the included studies are mostly cross-sectional, thus precluding the inference of causal relationships between oral health status and frailty. Secondly, older adults often have poor physical conditions, and the present study did not include the medical history of the analyzed patients, which could affect frailty. Thirdly, due to limitations in the number of studies for different outcomes, publication bias analysis was not performed in this study. Therefore, caution should be exercised when extrapolating the study conclusions. Further in-depth research is needed to validate our findings. Lastly, the included studies defined older adults differently, so caution should be exercised when extrapolating the results. In summary, large-scale, high-quality longitudinal studies are needed in the future to establish the causal relationship between oral health and frailty, thereby providing scientific support for geriatric care in clinical practice.

## Conclusion

This meta-analysis emphasizes the relationship between various oral health indicators and frailty, indicating that oral health status is likely intimately connected to the occurrence and development of frailty in older adults. Our study offers novel evidence-based support for the notion that oral health may increase the risk of frailty. Going forward, there should be a heightened emphasis on the oral health of seniors to avert the emergence and advancement of frailty, thereby safeguarding the health, longevity, and fulfillment of life for the growing aging population.

## Data Availability

The raw data supporting the conclusions of this article will be made available by the authors, without undue reservation.
