# Supplementary material for: Association between oral health status and frailty in older adults: a systematic review and meta-analysis
Source: Front Public Health. 2025 Mar 31;13:1514623. doi: 10.3389/fpubh.2025.1514623 (PMC11995049; doi:10.3389/fpubh.2025.1514623)
Supplement: Supplementary file 1 [file Table_1.DOCX]

**Table S1 The search strategy of meta-analysis**

| **Items** | **Contents** |
| --- | --- |
| Searchable electronic database | PubMed |
| PubMed search form | (((((((((((((((((((((((((((((((((((((((((((Carious Lesions[Title/Abstract]) OR (Carious Lesion[Title/Abstract])) OR (Lesion, Carious[Title/Abstract])) OR (Lesions, Carious[Title/Abstract])) OR (Carious Dentin[Title/Abstract])) OR (Carious Dentins[Title/Abstract])) OR (Dentin, Carious[Title/Abstract])) OR (Dentins, Carious[Title/Abstract])) OR (Root Caries[Title/Abstract])) OR (Caries, Root[Title/Abstract])) OR (Caries, Cervical[Title/Abstract])) OR (Cary, Cervical[Title/Abstract])) OR (Cervical Cary[Title/Abstract])) OR (Cervical Caries[Title/Abstract])) OR (Periodontal Diseases[Title/Abstract])) OR (Disease, Periodontal[Title/Abstract])) OR (Diseases, Periodontal[Title/Abstract])) OR (Periodontal Disease[Title/Abstract])) OR (Parodontosis[Title/Abstract])) OR (Parodontoses[Title/Abstract])) OR (Pyorrhea Alveolaris[Title/Abstract])) OR (Periodontitis[Title/Abstract])) OR (Periodontitides[Title/Abstract])) OR (Pericementitis[Title/Abstract])) OR (Pericementitides[Title/Abstract])) OR (Gingivitis[Title/Abstract])) OR (Gingivitides[Title/Abstract])) OR (Xerostomia[Title/Abstract])) OR (Xerostomias[Title/Abstract])) OR (Asialia[Title/Abstract])) OR (Asialias[Title/Abstract])) OR (Hyposalivation[Title/Abstract])) OR (Hyposalivations[Title/Abstract])) OR (Dentures[Title/Abstract])) OR (Denture[Title/Abstract])) OR (Toothbrushing[Title/Abstract])) OR (Toothbrushings[Title/Abstract])) OR (Dentifrices[Title/Abstract])) OR (Dentifrice[Title/Abstract])) OR (Toothpaste[Title/Abstract])) OR (Toothpastes[Title/Abstract])) OR (Mouthwashes[Title/Abstract])) OR (((((oral[Title/Abstract]) OR (mouth[Title/Abstract])) OR (Dental[Title/Abstract])) OR (Tooth[Title/Abstract])) OR (Teeth[Title/Abstract]))) AND (((((((Frailty[Title/Abstract]) OR (Frailties[Title/Abstract])) OR (Frailness[Title/Abstract])) OR (Frailty Syndrome[Title/Abstract])) OR (Debility[Title/Abstract])) OR (Debilities[Title/Abstract]))) |

**Table S2 Specific indicators of oral health status and their definitions**

| **Categorization** | **Indicators** | **Definitions** |
| --- | --- | --- |
| **Dental-related** |  |  |
|  | Number of teeth | Amount of natural teeth: 0-32. |
|  | Functional dentition  (≥ 21 teeth) | ≥ 21 teeth |
|  | No false teeth | - |
|  | ≤ 20 teeth, with denture | - |
| **Oral hygiene care** |  |  |
|  | Tooth brushing daily | Brush tooth once a day, twice a day, three times a day or more. |
| **Oral function** |  |  |
|  | Oral moisture | Oral moisture detectors are utilized to assess the hydration level of the mucosal surface on the dorsal surface of the tongue's midsection. |
|  | Oral dryness (< 27) | An oral moisture meter is employed to estimate the wetness of the mucosal surface on the dorsal aspect of the tongue's midsection, with a sensor value below 27.0 indicating xerostomia. |
|  | Tongue pressure (kPa) | - |
|  | Decreased tongue pressure (< 30 kPa) | - |
|  | Occlusal force/Occlusal force Reduced | After the exclusion of residual roots and loose teeth (three in number), a diagnosis of reduced occlusal force is made when the remaining number of teeth is less than 20 |
|  | Masticatory function (mg/dl) | The masticatory ability is assessed by measuring the glucose concentration after chewing a soft candy, with units in milligrams per deciliter (mg/dl). |
|  | ODK hypofunction | Oral function is assessed using the ODK measurement tool, which involves the rapid repetition of the monosyllabic sounds /pa/, /ta/, and /ka/ for 5 seconds. A diagnosis of reduced ODK function is made if at least one of the three syllables scores below 6. |
|  | Poor mixing ability | The assessment of masticatory function's mixing ability is conducted using color-changing chewing gum, with scores ranging from 1 (green-yellow, indicating the poorest mixing ability) to 5 (red, indicating the best mixing ability). A score of ≤3 is considered poor. |
| **Comprehensive Oral Health Score** |  |  |
|  | OHAT | The OHAT evaluates the comprehensive oral health status of participants. It assesses various aspects of oral health, including lips, tongue, gums and tissues, saliva, natural teeth, dentures, oral cleanliness, and toothache. The OHAT evaluation involves assigning a score of 0, 1, or 2 for each item based on observed conditions. The total OHAT score ranges from 0 to 16, with the following interpretations: i: 0-3 points indicate oral health that can be maintained through daily care; ii: 4-8 points indicate oral changes requiring monitoring: observed changes and notable vulnerabilities that need surveillance; iii: 9-16 points indicate poor oral health: a care plan is necessary, and professional advice from a dentist is recommended. |

Abbreviation: mg/dl: Milligrams per deciliter; kPa: Kilopascal; ODK: Oral diadochokinesis; OHAT: Oral health assessment Tool.

**Table S3 Frailty assessment criteria and their definitions or scoring rules**

| **Frailty assessment criteria** | **Definitions/scoring rules** |
| --- | --- |
| CHS | Participants exhibiting three or more positive criteria of weight loss, exhaustion, low physical activity, decreased walking speed, and handgrip strength are classified as ‘frail’. Those showing 1-2 positive criteria are considered ‘pre-frail’. Participants who do not exhibit any positive criteria are categorized as ‘robust’. |
| J-CHS | The assessment consists of five components: shrinking, slowness, weakness, debilitation, and low physical activity. Participants receive 1 point for each criterion met, and 0 points otherwise. A total score of 3 or more indicates frailty, a score of 1 or 2 suggests pre-frailty, and a score of 0 indicates non-frailty. |
| FRAIL scale | The FRAIL assessment comprises five items: 'How much time did you feel tired during the past 4 weeks?', 'Do you have any difficulty walking up 10 steps alone without resting and without aids?', 'Do you have any difficulty walking several hundred yards alone and without aids?', 'Do you report 5 or more illnesses out of 11 total illnesses?', and 'Did they report a weight decline of 5% or greater within the past 12 months?' The method of determining frailty is consistent with the CHS standards. |
| FI | FI refers to the proportion of potential health deficit indicators present in an individual at a given point in time, relative to all measured indicators. The variables selected for the FI encompass a multidimensional array of health variables, including physical, functional, psychological, and social aspects. It is generally considered that FI ≥ 0.25 indicates frailty in the elderly; FI < 0.12 indicates non-frail elderly, and FI ranging from 0.12 to 0.25 suggests a pre-frailty stage. |
| KCL | KCL consists of 25 yes/no questions, categorized into seven domains: activities of daily living (5 items), physical strength (5 items), emotions (5 items), memory (3 items), oral function (3 items), nutrition (2 items), and isolation (2 items). Frailty status is defined by excluding the three oral function questions. This approach allows for a more rigorous analysis of the relationship between the objective assessment results of oral function and frailty as evaluated by the KCL. Since the average score for oral function issues is 0.68, after excluding these items, we subtract 1 from the original classification, categorizing scores of 0-2 as robust, 3-6 as pre-frailty, and 7 or higher as frail. |
| GFI | GFI is a validated questionnaire from the Netherlands, encompassing 15 questions that address physical, cognitive, social, and psychological domains. Each question is scored as 0 (negative) or 1 (positive), with a total score ranging from 0 to 15. A GFI total score of 4 or higher is indicative of frailty. |

Abbreviation: CHS: Cardiovascular health study; J-CHS: Japanese version of the CHS; FRAIL: Fatigue, resistance, ambulation, illnesses, and loss of weight; FI: Frailty index; KCL: Kihon checklist; GFI: Groningen Frailty Indicator.

**Table S4 Quality assessment scale for cross-sectional studies**

| **Study** | **Q1** | **Q2** | **Q3** | **Q4** | **Q5** | **Q6** | **Q7** | **Q8** | **Number of entries that meet the criteria** |
| --- | --- | --- | --- | --- | --- | --- | --- | --- | --- |
| Xia 2024 | Y | Y | Y | N | Y | Y | Y | Y | 7 |
| Wibianty 2024 | Y | Y | Y | N | Y | N | Y | Y | 6 |
| Hong 2024 | Y | Y | Y | N | Y | N | Y | Y | 6 |
| Liu 2023 | Y | Y | Y | N | Y | Y | Y | Y | 7 |
| Cruz‑Moreira 2023 | Y | Y | Y | N | Y | Y | Y | Y | 7 |
| Tan 2022 | Y | Y | Y | N | Y | Y | Y | Y | 7 |
| Ohara 2022 | Y | Y | Y | N | Y | Y | Y | Y | 7 |
| Hakeem 2021 | Y | N | Y | N | Y | Y | Y | Y | 6 |
| Everaars 2021 | Y | N | Y | N | Y | Y | Y | Y | 6 |
| Zhang 2020 | Y | Y | Y | N | Y | Y | Y | Y | 7 |
| Valdez 2019 | Y | N | Y | N | Y | N | Y | Y | 5 |
| Hakeem 2020 | Y | Y | Y | Y | Y | Y | Y | Y | 8 |
| Satake 2019 | Y | N | Y | N | Y | Y | Y | Y | 6 |
| Hasegawa 2019 | Y | Y | Y | N | Y | N | Y | Y | 6 |
| Iwasaki 2018 | Y | N | Y | N | Y | Y | Y | Y | 6 |
| Watanabe 2017 | Y | Y | Y | N | Y | Y | Y | Y | 7 |
| Andrade 2013 | Y | N | Y | N | Y | Y | Y | Y | 6 |
| Castrejón-Pérez 2012 | Y | N | Y | N | Y | Y | Y | Y | 6 |
| Yoshida 2021 | Y | N | Y | N | Y | N | Y | Y | 5 |
| Nakamura 2021 | Y | N | Y | N | Y | Y | Y | Y | 6 |
| Watanabe 2020 | Y | Y | Y | N | Y | N | Y | Y | 6 |
| Shimazaki 2020 | Y | Y | Y | Y | Y | Y | Y | Y | 8 |
| Horibe 2018 | Y | Y | Y | Y | Y | N | Y | Y | 7 |
| Yamanashi 2017 | Y | Y | Y | N | Y | Y | Y | Y | 7 |
| Kimble 2022 | Y | N | Y | N | Y | Y | Y | Y | 6 |

Note: Numbers Q1-Q8 in heading signified: Q1: Were the criteria for inclusion in the sample clearly defined? Q2: Were the study subjects and the setting described in detail? Q3: Was the exposure measured in a valid and reliable way? Q4: Were objective, standard criteria used for measurement of the condition? Q5: Were confounding factors identified? Q6: Were strategies to deal with confounding factors stated? Q7: Were the outcomes measured in a valid and reliable way? Q8: Was appropriate statistical analysis used? Abbreviation: Y: yes；N: No; U: Unclear.

**Table S5 Quality assessment scale for cohort studies**

| **Study** | **Ⅰ** | **Ⅱ** | **Ⅲ** | **Ⅳ** | **Ⅴ** | **Ⅵ** | **Ⅶ** | **Ⅷ** | **Total** |
| --- | --- | --- | --- | --- | --- | --- | --- | --- | --- |
| Castrejón-Pérez 2017 | 1 | 1 | 1 | 1 | 0 | 1 | 0 | 1 | 6 |
| Zhang 2023 | 1 | 1 | 1 | 0 | 0 | 1 | 1 | 1 | 6 |
| Takeuchi 2022 | 1 | 1 | 1 | 1 | 0 | 1 | 1 | 1 | 7 |

Note: Numbers I-Ⅷ in heading signified: Ⅰ: Representatives of the exposed cohort; Ⅱ: Selection of the non-exposed cohort; Ⅲ: Ascertainment of exposure; Ⅳ: Demonstration that outcome of interest was present at the start of the study; Ⅴ: Comparability of cohorts on the basis of the design or analysis; Ⅵ: Assessment of the outcome; Ⅶ: Was follow-up long enough for outcomes to occur? Ⅷ: Adequacy of follow-up of cohorts.

**Table S6 Main research outcomes of the included articles**

| **Study** | **Key findings** |
| --- | --- |
| Xia 2024 | Multiple linear regression analysis showed a significant total relationship between the number of teeth (β: -0.359, 95%CI: -0.473--0.244, *P* < 0.001) and frailty. After adjusting for Mini Nutrition Assessment-Short Form scores, the relationship between the number of teeth and frailty remained significant (β: -0.327, 95%CI: -0.443--0.211, *P* < 0.001). Nutrition partially mediated the relationship between the number of teeth and frailty (indirect effect estimate = -0.0121, bootstrap 95%CI: -0.0151--0.0092; direct effect estimate = -0.0874, bootstrap 95%CI: -0.1086--0.0678) in the fully adjusted model. |
| Wibianty 2024 | A significant correlation between periodontal status and frailty in older people (*P* < 0.05). There were significant differences in plaque scores between frail and normal subject groups (*P* = 0.000), in the bleeding on probing between the frail and normal subject groups (*P* = 0.003), and in the number of teeth between frail and normal subject groups (*P =*0.011). |
| Hong 2024 | Patients with deteriorated oral health demonstrated a higher prevalence of frailty. |
| Liu 2023 | After controlling for all potential confounding factors, mouth changes requiring monitoring (OR: 2.10, 95%CI: 1.34-3.31, *P* = 0.001) and unhealthy mouth (OR: 2.55, 95%CI: 1.61-4.06, *P* < 0.001) were significantly associated with increased odds of frailty among older adults. Brushing teeth twice or more times a day was found to be significantly associated with a lower prevalence of frailty (OR: 0.50, 95%CI: 0.32-0.78, *P* = 0.002). Conversely, never brushing teeth was significantly associated with higher odds of frailty (OR: 1.74, 95%CI: 1.06-2.88, *P* = 0.030). |
| Cruz‑Moreira 2023 | The frequency of frailty was 2.06 times higher (95%CI: 1.30-3.29) in patients with oral hypofunction, and this association was maintained in women (OR: 2.18; 95%CI: 1.21-3.94). Reduced occlusal force and decreased swallowing function were items significantly associated with the presence of frailty (OR:1.95; 95%CI 1.18-3.22 and OR:2.11; 95%CI: 1.39-3.19, respectively). |
| Tan 2022 | Compared to edentulousness, having teeth was associated with higher odds of being robust or pre-frail. Denture-wearers compared with edentulous persons were more likely be robust or pre-frail. |
| Ohara 2022 | The proportion of individuals with a lower ODK increased. The SXI score was associated with physical frailty status (adjusted odds ratio for a one-point increase in SXI, 1.12; 95% confidence interval, 1.061.19). However, no difference was observed between the amount of resting saliva and severity of physical frailty. |
| Hakeem 2021 | For each additional tooth, the RR for frailty was 0.99 (95%CI: 0.98-0.99) in the fully adjusted model. Participants with moderate-severe periodontitis had 1.08 RR (95%CI: 1.02-1.14) for frailty index compared with participants with no periodontitis after adjusting for age, gender, and poor nutritional intake. The association lost significance in the fully adjusted model. |
| Everaars 2021 | A dental emergency visit (OR: 2.0, 95%CI: 1.33-3.02; OR: 1.58, 95%CI: 1.00-2.49), experiencing oral problems (OR: 2.07, 95%CI: 1.52-2.81; OR: 2.87, 95%CI: 2.07-3.99), and making dietary adaptations (OR: 2.66, 95%CI: 1.31-5.41; OR: 5.49, 95%CI: 3.01-10.01) were associated with being at risk for frailty and survey-based frailty respectively. |
| Zhang 2020 | Participants with no more than 20 teeth were associated with higher odds of being frail whether wearing dentures or not. Denture using could not help lower the odds of being prefrail or frail for older adults with fewer teeth |
| Valdez 2019 | The prevalence of periodontal disease was high regardless of their frailty stat. There were significant associations between frailty and dentition status (OR: 2.49, 95%CI: 1.17-5.30), and frailty and active coronal decayed surface (OR: 3.01, 95%CI: 1.50-6.08) but only ACDS remained significant after adjusting for confounders (adjusted OR: 2.46, CI: 1.17-5.18). There was no association between frailty and DSU and frailty and SROH. |
| Hakeem 2020 | The number of teeth, functional dentition, and self-rated oral health showed significant associations with frailty after adjusting for demographic and socioeconomic variables for both FP and FI. Adjusting for nutritional status attenuated the associations. |
| Satake 2019 | Multivariate logistic regression analysis revealed significant correlations between frailty and number of teeth (*P* = 0.032; OR: 0.963; 95%CI: 0.930-0.997), and tongue pressure (*P* = 0.029; OR: 0.956; 95%CI: 0.919-0.996). No correlations were found between frailty and the diadochokinetic syllables/pa/,/ta/, and/ka/ or periodontitis. |
| Hasegawa 2019 | The number of remaining teeth was significantly higher in the robust group than in the frail group (21.1 ± 0.6 vs 16.4 ± 2.3). The bacterial count was significantly smaller in the frail group than in the robust group, and there was no significant relationship between frailty and oral moisture. |
| Iwasaki 2018 | Objectively measured chewing ability was significantly associated with frailty (*P* < 0.01) |
| Watanabe 2017 | The number of present teeth, occlusal force, masseter muscle thickness, and ODK rate decreased with age. The frail group had significantly fewer present teeth (women aged ≥ 70), lower occlusal force (women aged ≥ 70; men aged ≥ 80), lower masseter muscle thickness, and lower ODK rate than the robust group. Multivariate analysis indicated that oral function was significantly associated with frailty. |
| Andrade 2013 | Elderly individuals with a need for dental prostheses were significantly more likely to be prefrail and frail. Participants with 20 or more teeth had a lower chance of being frail than edentulous individuals. |
| Castrejón-Pérez 2012 | Those who had a higher probability of being frail included those who rated their oral health worse than others (OR: 3.2), and those who did not use dental services (OR: 2.1). The number of remaining teeth was significantly higher in the robust group than in the frail group (11.8 ± 9.2 vs 8.3 ± 8.6, *P* < 0.001). |
| Yoshida 2021 | There was a significant relationship between oral hypofunction and deficit-accumulation model-assessed frailty, after adjusting for sarcopenia. |
| Nakamura 2021 | Rates of reduced occlusal force, decreased tongue pressure, decreased masticatory function and deterioration of swallowing function in the frailty group were significantly higher than those in the healthy group. In crude models, frailty was significantly associated with reduced occlusal force, and deterioration of the swallowing function. Further, in adjusted models including age, gender, BMI, education level, and physical activity level as covariates, frailty was independently associated with deterioration of swallowing function (OR: 2.56; 95%CI: 1.26-5.20). |
| Watanabe 2020 | After adjusting for confounders, the odds ratios of frailty comparing the highest to the lowest chewing gum score groups were 2.09 (95%CI: 1.09-4.03; *P* = 0.009). |
| Shimazaki 2020 | The multivariate OR for a pre-frail or frail status was significantly higher for older people with reduced occlusal force, reduced tongue–lip motor function, and deteriorated swallowing function than in those without deterioration of those items. Of the oral function items, swallowing function was most strongly associated with the frailty status, and the OR (95%CI) for deteriorated swallowing function in pre-frail and frail patients was 6.4 (3.9-10.8) and 10.2 (5.4-19.1), respectively. Those with oral hypofunction had significantly higher adjusted ORs for pre-frail (OR 1.4, 95%CI: 1.1-2.0) and frail (OR: 2.1, 95%CI: 1.2-3.5) statuses. |
| Horibe 2018 | Significant correlations of pre-frailty or frailty with maximum occlusal force, mixing ability, and subjective chewing ability were observed. |
| Yamanashi 2017 | In logistic regression analysis, increments in MIP were significantly associated with lower risk of frail and pre-frail status. The OR for frailty in relation to a 1-kPa increment in MIP was 0.90 (95%CI: 0.87-0.94, *P* < 0.001). The OR for frailty in relation to a 1-SD increment in MIP was 0.37 (95%CI: 0.26-0.54, *P* < 0.001). Even after multivariable adjustment for confounding factors, these associations remained significant. |
| Kimble 2022 | In the BRHS, but not HABC study, impaired natural dentition without the use of dentures was associated with frailty independently. This relationship was only established in the same group in those with oral problems (OR: 3.24; 95%CI: 1.30-8.03). In the HABC Study, functional dentition with oral health problems was associated with greater risk of frailty (OR: 2.21; 95%CI: 1.18-4.15). |
| Castrejón-Pérez 2017 | Each additional tooth was associated with a lower probability of developing frailty by 5.0% (RR: 0.90; 95%CI: 1.02-1.10). The 3-year risk ratio of developing frailty was 2.13 times higher (95%CI: 1.01-4.50) among participants having severe periodontitis. |
| Zhang 2023 | Age, number of natural teeth, use of dentures, toothache, and sex were independent risk factors for frailty at baseline. After full adjustment, multivariate logistic regression analysis indicated that compared with having ≥ 21 teeth, edentulism (OR: 3.575; 95%CI: 2.095-6.101) and partial tooth loss (OR: 2.448; 95%CI: 1.592-3.766) were associated with progression to frailty. Compared with those with ≥ 21 teeth, those with < 21 teeth and without dentures (OR: 2.617; 95%CI: 1.713-3.999) were more likely to progress to frailty. |
| Takeuchi 2022 | The newly identified frailty group at follow-up showed significantly lower values in the number of teeth present, ODK/ta/sound and ODK/ka/sound rates, and clinical attachment level at baseline compared to the robust group. A logistic regression model showed a significantly negative association between the ODK/ta/sound rate at baseline and the incidence of frailty. Articulatory oral motor skill was found to be a predictor of frailty after two years. |

Note: ACDS: Active coronal decayed surface; BMI: Body mass index; BRHS: British Regional Heart Study; DSU: Dental service utilization; FI: Frailty index; FP: Frailty phenotype; HABC: Health, Aging, and Body Composition; MIP: Maximum isometric tongue pressure; ODK: Oral diadochokinesis; OR: Odds ratio; RR: Rate ratio; SD: Standard deviation; SRH: Self-rated oral health SXI: Summated Xerostomia Inventory.

**Table S7 Sensitivity analyses for all associations**

| **Categorization** | **Indicators** | **SMD/OR (95% CI)** |
| --- | --- | --- |
| **Dental-related** |  |  |
|  | Number of teeth | -0.591 (-0.772, -0.411) |
|  | Functional dentition  (≥ 21 teeth) | 0.236 (0.162, 0.344) |
|  | No false teeth | 0.733 (0.538, 1.000) |
|  | ≤ 20 teeth, with denture | 2.320 (1.703, 3.160) |
| **Oral hygiene care** |  |  |
|  | Tooth brushing daily | 0.562 (0.396, 0.797) |
|  |  |  |
| **Oral function** |  |  |
|  | Oral moisture | 0.214 (-0.067, 0.495) |
|  | Oral dryness (< 27) | 1.141 (0.594, 2.192) |
|  | Tongue pressure (kPa) | -0.582 (-1.023, -0.141) |
|  | Decreased tongue pressure (< 30 kPa) | 1.618 (1.116, 2.346) |
|  | Occlusal force (N) | -0.526 (-0.808, -0.245) |
|  | Occlusal force Reduced | 1.846 (1.208, 2.820) |
|  | Masticatory function (mg/dl) | -0.442 (-1.026, 0.142) |
|  | ODK hypofunction | 1.876 (1.334, 2.639) |
|  | Poor mixing ability | 2.303 (1.692, 3.134) |
| **Comprehensive Oral Health Score** |  |  |
|  | OHAT scores ≥ 4 | 2.501 (1.752, 3.570) |

Abbreviation: CI: Confidence interval; mg/dl: Milligrams per deciliter; kPa: Kilopascal; ODK: Oral diadochokinesis; OHAT: Oral health assessment Tool; OR: Odds ratio; SMD: Standardized mean difference.
